# Chemotherapy induced right ventricular cardiomyopathy; a systematic review and meta-analysis

**DOI:** 10.3389/fcvm.2023.1103941

**Published:** 2023-08-03

**Authors:** Pramod Theetha Kariyanna, Ashish Kumar, Amog Jayarangaiah, Mrinali Shetty, Yuvraj Chowdhury, Sushruth Das, Apoorva Jayarangaiah

**Affiliations:** ^1^ Department of Cardiology, Chaparral Medical Group/Pomona Valley Hospital Medical Center, Pomona, CA, United States; ^2^ Department of Internal Medicine, Cleveland Clinic Akron General, Akron, OH, United States; ^3^ Department of Internal Medicine, Marshfield Clinic, Marshfield, WI, United States; ^4^ Department of Cardiology, New York Presbyterian Hospital (Columbia and Cornell Campus), New York, NY, United States; ^5^ Department of Cardiology, University of Massachusetts, Boston, MA, United States; ^6^ Avalon University School of Medicine, Willemstad, Curaçao; ^7^ Department of Hematology/Oncology, Prevea Clinic/HSHS Sacred Heart, Eau Claire, WI, United States

**Keywords:** right ventri cular dysfunction, anthracycline, trastuzumab, cardiomyopathy, chemotherapy, breast cancer, cardiooncology

## Abstract

**Background:**

Left ventricular dysfunction and cardiomyopathy are well documented adverse effects associated with chemotherapy agents. Limited information exists regarding the impact of chemotherapeutic agents on the integrity and function of the right ventricle (RV).

**Objectives:**

The current metanalysis compared pre- chemotherapy versus post- chemotherapy RV parameters measured on 2D echocardiography in patients receiving anthracycline and/or trastuzumab across all breast cancer patients.

**Methods:**

A systematic search across PubMed, EMBASE and Cochrane databases were performed from inception of the databases until November 2021 for relevant studies. We used the inverse variance method with a random effect model and DerSimonian and Laird method of Tau2 generation to calculate mean difference [MD] with 95% confidence interval [CI]. The analysis was carried out using RevMan Version 5.3 (Copenhagen: The Nordic Cochrane Centre, The Cochrane Collaboration, 2014).

**Results:**

Fifteen studies, constituting total of 644 patients, met the inclusion criteria, with most studies having a follow up period of less than 12 months from initiation of chemotherapy. Anthracycline and/or Trastuzumab chemotherapy resulted in a statistically significant reduction in right ventricular ejection fraction (RVEF) at follow-up [MD: 2.70, 95% CI: 0.27 to 5.13, *P*-value- 0.03, *I*^2^- 71%, *χ*^2^ *P*-value < 0.05]. Treatment with Anthracycline and/or Trastuzumab chemotherapy resulted in a significant reduction in RV fractional area change (RVFAC) at follow-up [MD: 3.74, 95% CI: 1.33 to 6.15, *P*-value < 0.01, *I*^2^- 68%, *χ*^2^ *P*-value < 0.05]. RV free wall longitudinal strain (RVFWLS) was lower at baseline, while LVEF was significantly reduced at follow-up [MD: -1.00, 95% CI: -1.86 to -0.15, *P*-value < 0.05, *I*^2^- 0%, *χ*^2^ *P*-value-0.40], [MD: 4.04, 95% CI: 2.08 to 6.01, *P*-value < 0.01, *I*^2^- 91%, *χ*^2^ *P*-value < 0.05], respectively. However, treatment with Anthracycline and/or Trastuzumab chemotherapy had no statistically significant effect on Tricuspid annular plane systolic excursion (TAPSE) at follow-up [MD: 0.53, 95% CI: -0.11 to 1.17, *P*-value-0.11, *I*^2^- 98%, *χ*^2^ *P*-value < 0.05].

**Conclusions:**

Chemotherapy with anthracyclines and trastuzumab negatively affects right ventricular function leading to decline in RVEF, RVFAC, RVFWLS and LVEF.

## Introduction

Cancer specific mortality rates have substantially declined over the past few decades owing to the diagnostic and therapeutic advances within the field. However, the adverse effects of cardiotoxicity of specific chemotherapeutic drugs, namely anthracyclines and trastuzumab continue to contribute to significant morbidity and mortality in this patient population. Deducing the adverse effects of these chemotherapeutic drugs on the cardiovascular system is pivotal due the presence of heart failure from chemotherapy-induced cardiomyopathy and premature coronary artery disease among a considerable number of patients (1). The first such effect was recognized in the 1960s with the advent of Daunorubicin, an anthracycline, which gave rise to the niche sub-specialty known as “Cardio-Oncology” (2–4). The increasing awareness and poor prognosis associated with chemotherapy-induced cardiomyopathy has ushered the development of protocols for screening, surveillance as well as interventions to reduce cardiac risk (5–8). The field continues to evolve to better identify, define predictive risk models and biomarkers of cancer therapy related cardiac dysfunction (CTRCD).

Left ventricular dysfunction and remodelling has been well defined in chemotherapy induced cardiotoxicity. The effect of chemotherapeutic agents on the right ventricle (RV) is not well researched. Several initial reports on CTRCD have demonstrated right ventricle involvement following chemotherapy, as seen on right ventricular biopsies (9). Nevertheless, there is a paucity of data with respect to the frequency of RV involvement and its prognostic value in CTRCD. Echocardiographic evaluation of the RV should include RV basal diameter, right atrial size (area), tricuspid annular plane systolic excursion via M-mode, tricuspid annular systolic peak velocity (TAPSE) using pulsed doppler tissue imaging (DTI), RV fractional area shortening (FAC) and provide estimates of the RV systolic pressure. Individual studies assessing the effects of cancer chemotherapeutics on the RV exist, but they are limited by a small patient cohort. In the current systematic review and meta-analysis we report the pooled differences in RV echo parameters pre and post anthracycline and/or trastuzumab across all breast cancer patients.

## Methods

### Data sources and search strategies

A systematic search across PubMed, EMBASE and Cochrane databases were performed from inception of the databases until November 2021 for relevant studies. Supplementary file provides the search strategy and MeSH terms used in each database (Supplementary Table S1). In addition to database search, we also reviewed the reference lists of included articles to identify additional studies.

The present analysis was performed according to the Preferred Reporting Items for Systematic Reviews and Meta-Analyses (PRISMA) and American Heart Association guidelines (10, 11). Since, only studies with prior ethical committee clearance were included in the present analysis, no separate ethical clearance was required.

### Study selection

After checking for duplicates, the searched articles were screened for relevant studies. The inclusion criteria were as follows: (a) observational studies, reporting right ventricular echocardiography outcomes at baseline and post Anthracycline and/or Trastuzumab chemotherapy among breast cancer patients; (b) no exclusion based on sample size; (c) reporting one of the following right ventricular echocardiography outcomes; right ventricle ejection fraction (RVEF), right ventricular fractional area change (RVFAC), right ventricular global longitudinal strain (RVGLS), right ventricular free wall longitudinal strain (RVFWLS), tricuspid annular plane systolic excursion (TAPSE); (d) exclusion of studies conducted exclusively in hematologic malignancies and pediatric cancer survivors and (e) finally, studies published in English language. The screening for inclusion was performed independently by two reviewers (A.J., M.S.) at two separate levels. At level one, title and abstracts of searched citations were screened for relevance. At the second level, articles identified from level one screening were subjected to complete manuscript review and considered when they met the inclusion criteria.

### Screening and data extraction

Data extraction from included studies was performed independently by two reviewers (A.J., M.S.), and later examined for similarity. The following information was extracted from original primary publications; author, year of study, sample size, diagnosis, cancer chemotherapy agent used, percentage of patients treated with Anthracyclines, follow-up period, baseline and follow-up values of RVEF, RVFAC, RVGLS, RVFWLS, TAPSE, and left ventricular ejection fraction (LVEF). The data extraction was performed as per a pre-designed data extraction form. Any disparity was resolved by mutual consensus and after consultation with other authors. Studies included observational studies without any interventions, therefore bias tools such as the ROBINS-I tool were not employed. The National Institute of Health (NIH) Quality Assessment Tool for Before-After (Pre-Post) Studies with No Control Group was utilized for risk of bias assessment in the current analysis.

### Quality assessment

We used the National Institutes of Health (NIH) Quality Assessment Tool for Before-After (Pre-Post) Studies with No Control Group. It included the following assessments; pre-specified entry criteria, sample size, blinded assessors of data and clear descriptors of outcome measurements and others. Two authors independently judged each domain, with a third author resolving conflicts.

### Statistical analysis

We used the inverse variance method with a random effect model and DerSimonian and Laird method of Tau2 generation to calculate mean difference [MD] with 95% confidence interval [CI]. We used the method provided by Hozo et al., to calculate mean and standard deviation from studies reporting median and inter quartile range (12). To obtain a conservative result with a wide confidence interval, we used a correction (*r* = 0) between the pre-treatment and post treatment mean while calculating the standard deviation of the pooled estimate. Statistical heterogeneity was assessed using Higgins's *I*^2^ or chi-square *P*-value (13). The pooled estimate was deemed statistically heterogeneous if Higgins's *I*^2^ was >50% or chi-square *P*-value was < 0.05. Meta-analysis results were reported graphically using forest plots: the measure of effect (MD) was represented by a square, with the area being proportional to study weight. A *p* value < 0.05 was considered significant. The analysis was carried out using RevMan Version 5.3 (Copenhagen: The Nordic Cochrane Centre, The Cochrane Collaboration, 2014).

## Results

### Study selection

**Figure 1:**
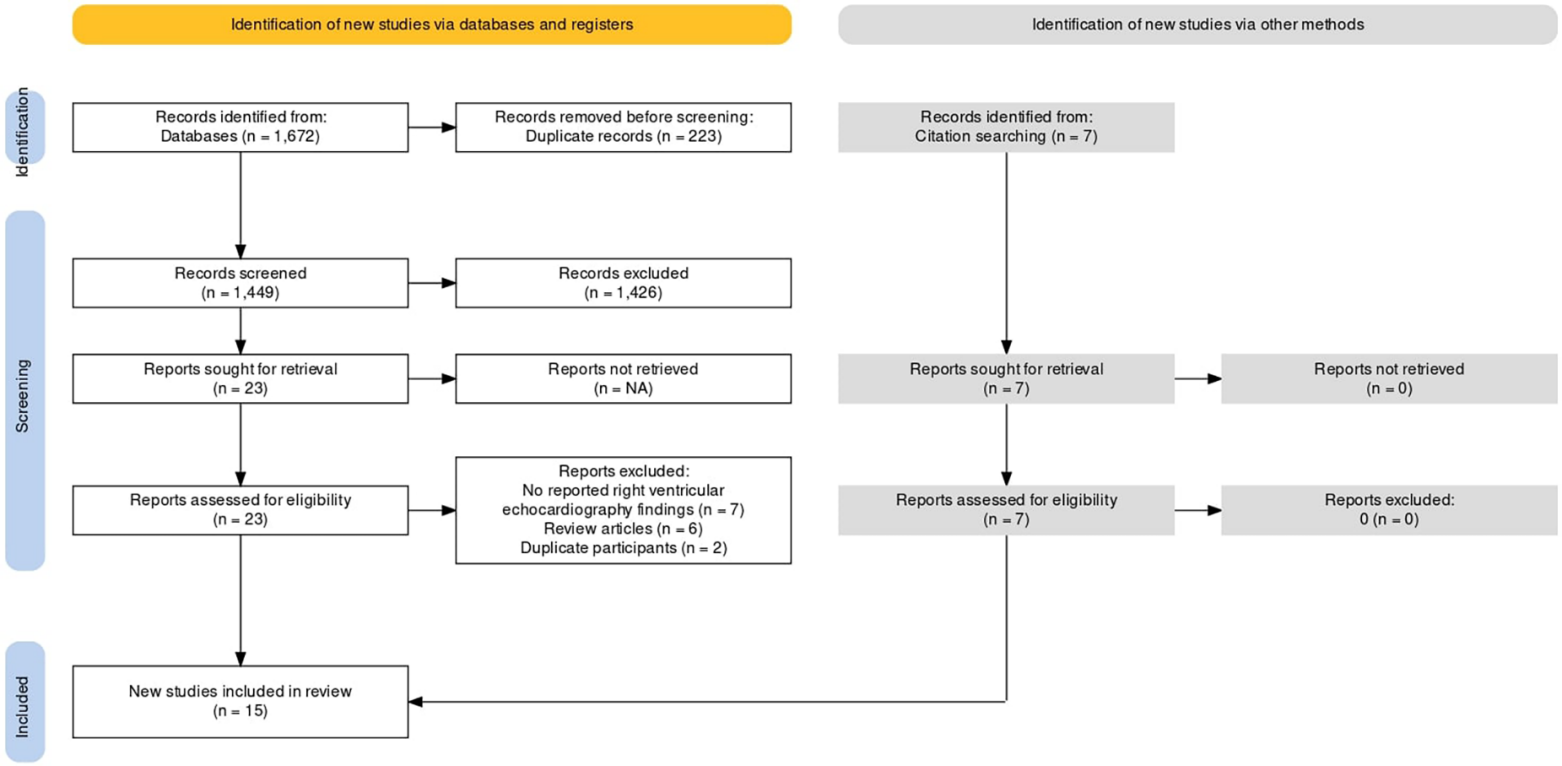
PRISMA flow chart.

The database search identified a total of 1,449 articles after checking for duplicates. Figure 1 represents the PRISMA flow chart for the inclusion of studies. 1,426 studies were excluded at the first level of screening and 15 studies were excluded at the second level of screening. Seven studies were included after manual search of reference list of the included studies. Finally, fifteen studies met the inclusion criteria and were included in the final analysis (14, 15, 24–26, 16–23). The fifteen studies constituted a total of 644 patients. All studies included breast cancer patients who were treated with Anthracycline and/or Trastuzumab chemotherapy, other than study by Cottin et al., 1996 which included women with breast cancer and lymphoma. Follow-up period of all included studies was less than 12 months. Baseline characteristics of patients from the included studies are presented in Table 1. We included studies which assessed right ventricular parameters by 2D echo parameters including RVEF, RVFAC, RVGLS, RVFWLS, TAPSE, as defined by the American Society of Echocardiography definitions and reference ranges for each parameter in Table 2. Summaries of individual studies included in the present systematic review and meta-analysis are presented in Table 3.

**Table 1:** Baseline characteristics of included studies.

Author	Year	*N*	Primary Site	Chemotherapy And/or Monoclonal Antibody	Cumulative anthracycline dose used (median) mg/m^2^	Percentage of patients treated with anthracyclines	RV parameter evaluated/Modality	Follow up period
Havsteen et al.	1989	14	Breast	Anthracycline (Epirubicin)	827	100%	RVEF	2-3 weeks after the last chemotherapy
Cottin et al.	1996	33	Breast + Lymphoma	Anthracycline	The doses ranged from 100 to 550 mg/m^2^ of body surface area (mean: 260_+92 mg/m^2^).	100%	RVEF PER TPER PFR TPFR	1 month and 12 months
Grover et al.	2013	46	Breast	Anthracycline + Traztuzumab	Epirubicin 100 mg/m^2^ (3-6 cycles) Doxorubicin 50 m/m^2^ (3–6 cycles)	67%	RVEF RVEDVI RVESVI	4 months and 12 months
Calleja et al. #	2015	30	Breast	Anthracycline +/−Trastuzumab	Epirubicin = 302.4 Doxorubicin = 231.2	73%	RVSP RV FAC RV FWLS RV GLS	Time to cardiotoxicity 5.0 months (A+) 7.5 months (A-) Follow up period 23 months
Nakano et al.	2016	9	Breast	Trastuzumab +/−Anthracycline	–	66.7%	RV FWPSCS RV LS RVEF	3, 6, 12 months
Barthur et al.	2017	41	Breast	Trastuzumab +/−Anthracycline	–	56.1%	RVEF RVEDV RVESV	6,12, 18 months
Tanindi et al.	2011	37	Breast	Anthracycline	60 mg/m^2^ × 6 cycles = 360	–	RVFAC TAPSE	Echocardiography was performed before the onset of the chemotheurapeutic regimen (T1), on the day after the completion of the first cure (T2), and after the completion of two cures of the regimen (T3).
Moustafa et. al	2015	50	Breast	Trastuzumab +/−Anthracyclines	<240 mg/m^2^	28%	RVFAC TAPSE RVSP RVGLS	12–15 months
Boczar et al.	2016	49	Breast	Anthracycline	–(*N* = 15)doxorubicin was 232 –(*N* = 34)fluorouracil/epirubicin/cyclophosphamide every 3weeks × 3 ± docetaxel was 294.12	100%	RV FAC RV FWLS	Mean: 125 days; 95% CI: 107–142days
Esfahani et al.	2016	49	Breast	Anthracycline	450–550	100%	RVFAC RVEDD	6 months
Chang et al.	2016	35	Breast	Epirubicin	354.19 ± 336.08	100%	RVFAC RV FWLS	21, 42 and 63 days
Arciniegas et al.	2018	66	Breast	Trastuzumab + Anthracycline (doxorubicin and epirubicin)	anthracycline dose of 252 (±45)	100%	RVLS RVGCS RVGLS RVGCSRs	Till second echo after chemo initiation. Median 5.4 months
Keramida et al.	2019	101	Breast	Trastuzumab (Also Anthracycline And Taxanes)	Mean dose 483 (±145.1) mg/kg	61.4%	RVGLS RV FWLS	12 months
Lange et al.	2012	42	Breast	Trastuzumab +/−Anthracyclines	–	73.8%	RVSP RV diameter	3 and 6 months
Kilicasian et al.	2015	42	Breast	Trastuzumab and Anthracyclines	–	33%	RV MPI TAPSE	6 months

^#^ 30 cases and age and cardiac risk factor balanced women with a diagnosis of HER2 + breast cancer without any prior cardiac history and who had an echocardiogram prior to initiation of any cancer therapy were included as controls, since the cases didn't have a baseline echocardiogram. RV, Right ventricle; PER, peak ejection rate; PFR, peak filling rate; TPER, time to peak ejection rate; TPFR, time to peak filling rate; TAPSE, tricuspid annular plane systolic excursion; RVFWPSCS, right ventricular (RV) free wall peak systolic circumferential strains; RVLS, right ventricular longitudinal strain; RVFAC, right ventricular fractional area change; RVEDVI, right ventricular end-diastolic volume index; RVESVI, right ventricular end-systolic volume index; RV FWLS, right ventricular free wall longitudinal strain; RV GLS, right ventricular global longitudinal strain; RVGCS, right ventricular global circumferential strain; RVGCSRs, right ventricular global circumferential systolic strain rate; RVEDD, right ventricle end-diastolic diameter; RVSP, Right ventricular Systolic pressure; RV MPI, right ventricular myocardial performance index.

**Table 2:** Description of common parameters used to assess right ventricular function as defined by the American society of echocardiography.

RV Parameter	Definition	Reference Range	What does it assess?
RVEF	End diastolic volume- end systolic volume/end diastolic volume	45%	RV systolic function
RVFAC	End diastolic area-end systolic area/end diastolic area × 100	35% and above	Measure of RV systolic function Measures both longitudinal and radial components of RV
Strain: Strain is defined as percentage change in myocardial deformation			
RVFWLS	Change in length in RV free wall over baseline length	<−20%	Assesses regional RV systolic function
RVGLS	Global change in length over baseline length	<−20%	Assesses global RV systolic function
TAPSE	A method used to measure the distance of systolic excursion of the RV annular segment along its longitudinal plane, from a standard apical 4-chamber window.	>16 mm	RV longitudinal function

**Table 3:** Summaries of individual studies included in the current systematic review and meta-analysis.

Author	Year	Mean Age (years)	Region	Drug	Radiation	Inclusion criteria	Conclusion
Havsteen et al.	1989	53 (34–66)	Denmark	Epirubicin	–	Female breast cancer patients epirubicin compared to female breast cancer patients treated with cyclophosphamide, methotrexate and 5-fluorouracil	Treatment with epirubicin resulted in a significant decrease in LVEF at rest and during exercise compared to the CMF treatment in patients with advanced breast cancer. RVEF was unaffected. 1 patient developed fatal CHF
Cottin et al.	1996	51+/–13	France	Anthracycline	–	Women treated with Anthracycline chemotherapy	The study found a significant decrease in LVEF, and LV diastolic function after chemotherapy. There were no significant changes in RV parameters.
Grover et al.	2013	55+/–10	Australia	Anthracycline And/or traztuzumab	–	Women with breast cancer treated with anthracycline (3–6 cycles) and/or traztuzumab	The study found significant decreases in both the LVEF and RVEF following chemotherapy.
Calleja et al.	2015	54+/−12	Canada	Anthracyclines +/−Traztuzumab	–	Women > 18 years With HER-2 + breastcancer at any stage treated with Traztuzumab + −anthracyclines. Who developed cardiotoxicity during treatment	The study showed Reduced RV-GLS and RV-FWLS in breast cancer patients with cardiotoxicity. These outcomes were compared with age matched breast cancer patients without cardiotoxicity
Nakano et al.	2016	62.3+/−12.6	Japan	Traztuzumab +/−Anthracylcines	55.6%	Female patients newly diagnosed with HER-2 + breast cancer.	The study found a a decrease in LVEF. RV-FWLS and ejection fraction remained unchanged.
Barthur et al.	2017	52+/−11	Canada	Traztuzumab +/−Anthracylcines	12%	Female patients > 18 years diagnosed with HER-2 + breast cancer with an LVEF > = 50% at baseline	Significant decreases in both the LVEF and RVEF following chemotherapy in breast cancer patients. In addition to significant increase in RV- end diastolic volume and RV-end systolic volume
Tanindi et al.	2011	41.9+/–5.2	Turkey	Anthracyclines	–	Female patients <50years, diagnosed with breast cancer treated with anthracylines that had no evidence of CAD or CAD risk factors	The study showed a decrease in RV FAC and RV TAPSE. LV systolic was not significantly affected
Moustafa et. al	2015	60+/−13	USA	Anthracylines +/−traztuzumab	62%	Female patients diagnosed with HER-2 + breast cancer treated with traztumab+/- anthracyclines	The study showed no significant change in LVEF and RVEF. However there was significant decrease in RVGLS
Boczar et al.	2016	53.4+/–3.3	Canada	Anthracyclines	–	Female patients with early stage HER-2 - breast cancer	The study showed a decrease in LVEF and a significant worsening in RV-FAC and RV-FWLS
Esfahani et al.	2016	40.75+/–7.14	Iran	Anthracyclines	–	Female patients <50Yrs with breast cancer, no prior CAD and LVEF >50%	The study showed a decrease in LVEF in addition to RV end-diastolic diameter increase, RV-FAC decrease and Decrease in TAPSE
Chang et al.	2016	45.33+/–8.48	Taiwan	Traztuzumab	10%	Female patients with breast cancer, LVEF >50%. With age and gender matched control populations	The study found that RV-FWLS significantly declined in addition to decline in TAPSE. No decline in LVEF.
Arciniegas et al.	2018	52+/–9	USA	Anthracylines +/−traztuzumab	76%	Female patients with newly diagnosed breast cancer. LVEF >50%	The study showed a decrease in LVEF. In addition to a decrease in RV GLS was seen, which had predictive value in determining cardiotoxicity
Keramida et al.	2019	54.3+/–11.4	USA	Anthracyclines	55%	Female patients with breast cancer, HER-2+	The study showed a decrease in LVEF, LVGLS and RVGLS
Lange et al.	2012	56 (38–75)	Germany	trastuzumab+/− Anthracyclines	–	Female patients	The study showed a worsening diastolic LV filling, increased E Vmax, altered tissue Doppler velocities, and decreased systolic function. All Right heart parameters showed no significant change
Kilicasian et al.	2015	50.4+/–11.6	Turkey	trastuzumab+/− Anthracyclines	31%	Female patients with HER 2 + breast cancer and LVEF >60%	The study showed a decrease in LVEF and TAPSE

Per the NIH Quality Assessment Tool for Before-After (Pre-Post) Studies with No Control Group, all the included studies had sample size not sufficiently large to provide confidence in the findings, and people assessing the outcomes were not blinded to the participants’ exposures/interventions, hence potentially leading to bias in the reported results.

**Figure 2:**
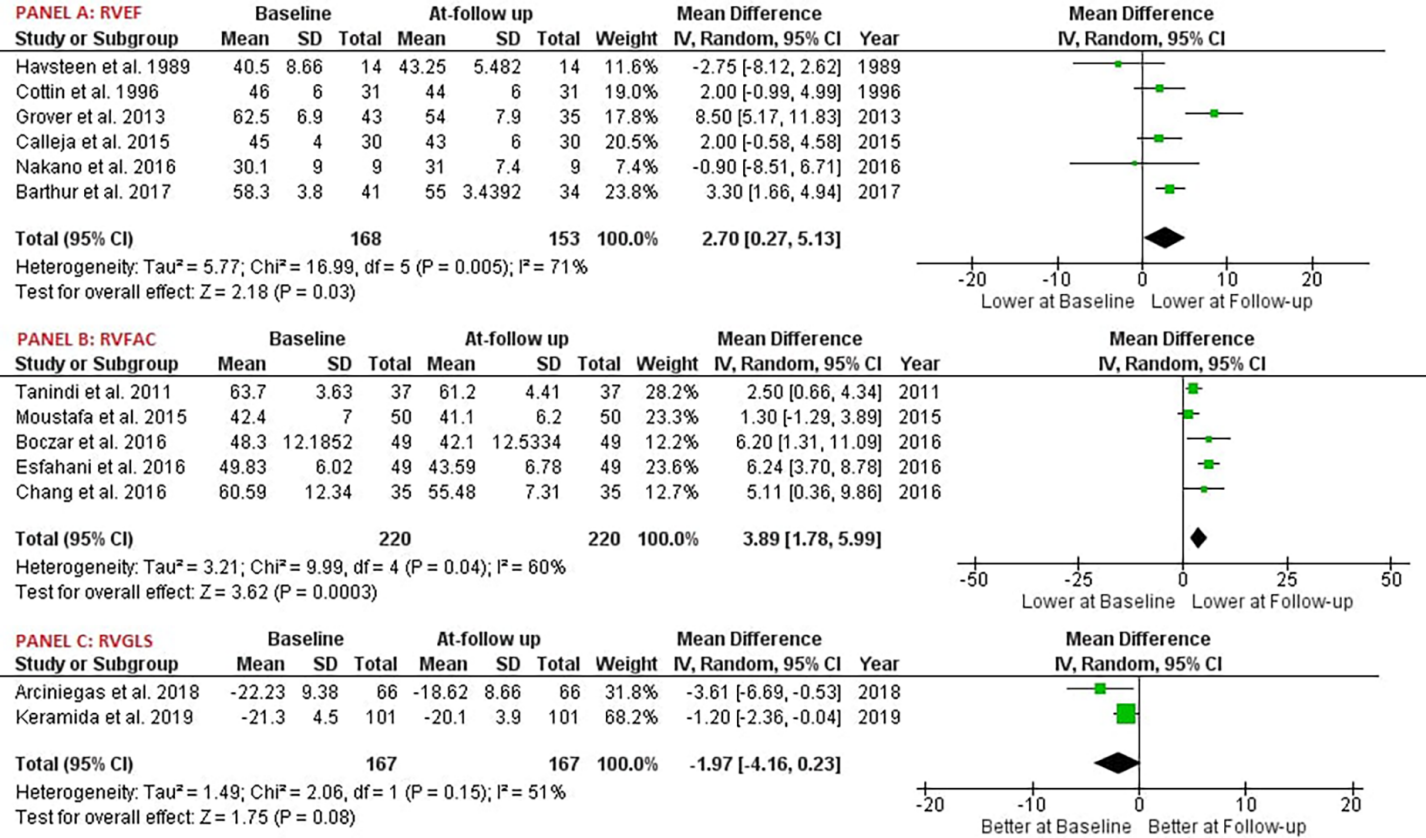
Forest plot; RVEF, right ventricular ejection fraction; RVFAC, right ventricular fractional area change; RVGLS, right ventricular global longitudinal strain.

**Figure 3:**
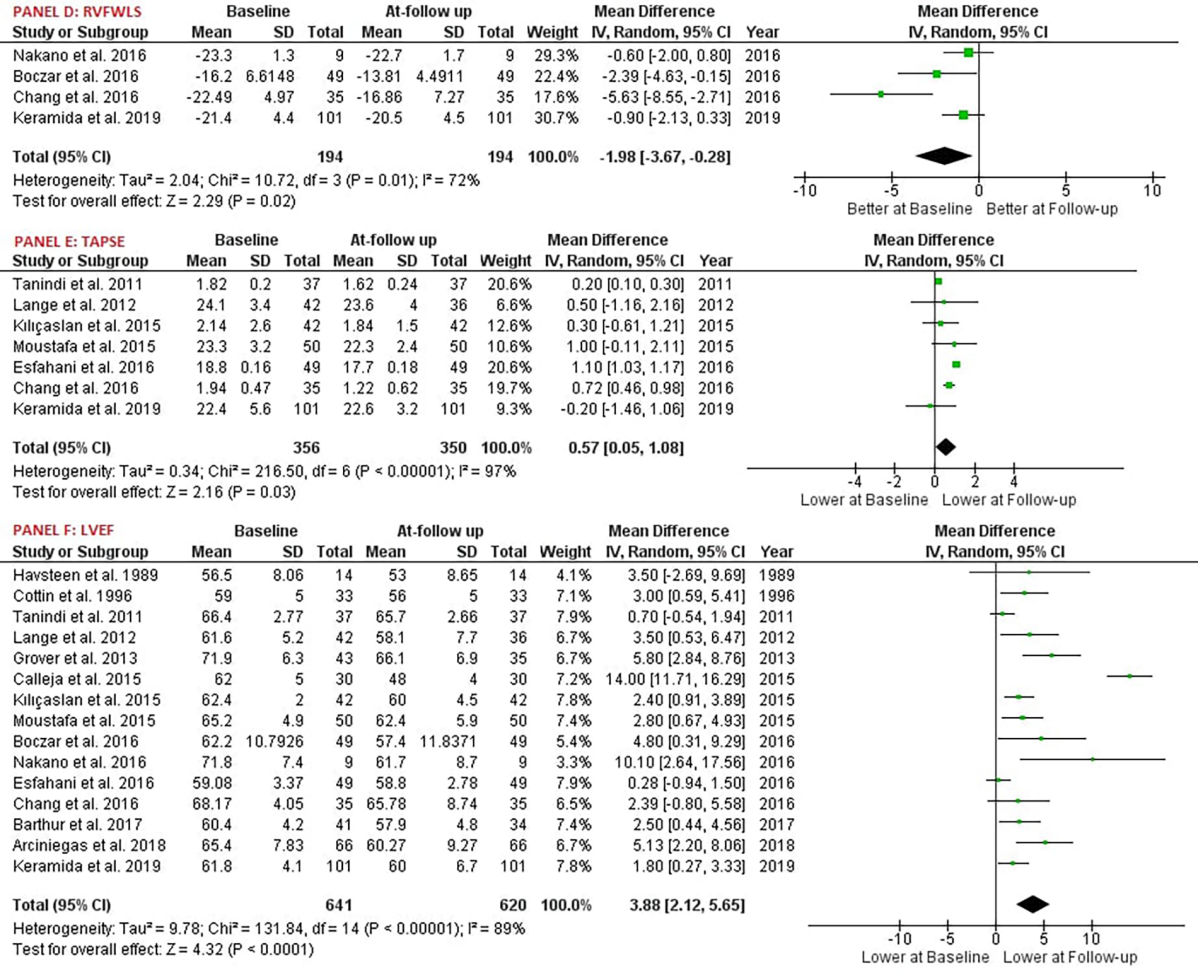
Forest plot; RVFWLS, right ventricular free wall longitudinal strain; TAPSE, tricuspid annular plane systolic excursion; LVEF, left ventricular ejection fraction.

Our study reported significant findings. Anthracycline and/or Trastuzumab chemotherapy led to a statistically significant decrease in Right Ventricular Ejection Fraction (RVEF), with six studies involving 168 patients providing data on RVEF [MD: 2.70, 95% CI: 0.27 to 5.13, *P* = 0.03, *I*^2^ = 71%, *χ*2 *P* < 0.05] (Figure 2A). Additionally, Right Ventricular Fractional Area of Change (RVFAC) decreased significantly following the chemotherapy treatment with Anthracycline and/or Trastuzumab, as depicted in five studies [MD: 3.74, 95% CI: 1.33 to 6.15, *P* < 0.01, *I*^2^ = 68%, *χ*2 *P* < 0.05] (Figure 2B). Chemotherapy with Anthracycline and/or Trastuzumab had no effect on Right Ventricular global longitudinal strain (RVGLS) [MD: -1.97, 95% CI: -4.16 to 0.23, *P* = 0.08, *I*^2^ = 51%, *χ*2 *P* = 0.15] (Figure 2C). Right Ventricular free wall longitudinal strain (RVFWLS) declined at follow-up, [MD: -1.00, 95% CI: -1.86 to -0.15, *P* < 0.05, *I*^2^ = 0%, *χ*2 *P* = 0.40] (Figure 3D). Furthermore, Left Ventricular Ejection Fraction (LVEF) was also reduced at follow-up [MD: 4.04, 95% CI: 2.08 to 6.01, *P* < 0.01, *I*^2^ = 91%, *χ*2 *P* < 0.05] (Figure 3F). Tricuspic annular plane systolic excursion (TAPSE) was not affected by the treatment [MD: 0.53, 95% CI: -0.11 to 1.17, *P* = 0.11, *I*^2^ = 98%, *χ*2 *P* < 0.05] (Figure 3E). However, most of the pooled estimates were associated with significant statistical heterogeneity, mostly because of the heterogeneity in the magnitude of effect measures rather than variation in effect of chemotherapy with Anthracycline and/or Trastuzumab on echocardiography parameters.

## Discussion

The present meta-analysis analysed the echocardiographic changes of RV following treatment with Anthracycline and/or Trastuzumab. This is one of the first studies with a large sample size of 644 patients to study the effects of chemotherapeutic agents on the RV, thereby, increasing its statistical power.

The two most commonly used chemotherapeutic agents associated with cardiotoxicity are Doxorubicin (an Anthracycline) and Trastuzumab (a recombinant DNA derived humanized monoclonal antibody targeting human epidermal growth receptor 2 (HER 2) (28). Doxorubicin associated cardiotoxicity is cumulative dose-dependent and has been attributed to an upsurge in inflammation, oxidative stress, dysregulation of autophagy, mitochondrial dysfunction and apoptosis (29). Trastuzumab causes asymptomatic decrease in LVEF and less frequently, overt congestive heart failure (30–32). These effects are not dose dependent and are reversible. Trastuzumab can potentiate the cardiac side-effects of Anthracyclines. The risk of cardiotoxicity with Trastuzumab monotherapy is 3%–7% as compared to 27% when combined with Anthracyclines (33).

Cancer Therapy-Related Cardiac Dysfunction (CTRCD) has been classified as Type I and Type II. Type I CTRCD encompasses Anthracycline-induced cardiotoxicity which begins at the time of drug initiation and progresses algorithmically with increase in cumulative dose (34). Once a threshold maximum is achieved, myofibrillar disarray and cardiac myocyte apoptosis ensues, manifesting as left ventricular dysfunction and irreversible cardiac injury. Type II CTRCD is associated with, but not unique to, Trastuzumab. There is loss of myocardial contractility, likely due to the phenomena of myocyte hibernation or stunning without cell death. This effect is reversible and less likely to be associated with overt congestive heart failure (35).

There are no existing evidence-based guidelines for the screening and surveillance of CTRCD, however, expert consensus has been published by several committees (36, 37). Baseline cardiac imaging, typically with 2D echocardiography, is obtained with the main aim of evaluating left ventricular function prior to initiation of Anthracyclines or HER-2 inhibitors. The modified biplane Simpson's technique is the method of choice for assessment of left ventricular function. If available, global longitudinal strain should also be employed. Although, multigated acquisition (MUGA) scan has consistently outperformed standard 2D echocardiography in quantifying LVEF measurements, it is not a good assessment tool for right ventricular function, valvular dysfunction, atrial enlargement or pericardial disease and is used as an adjunct to echocardiography (38, 39). Cardiac magnetic resonance imaging (CMR) is the reference standard for assessing ventricular volume and function. The ASE defined CTRCD as ⩾10% decline in LVEF to a final value less than 53% confirmed on subsequent imaging performed 2 to 3 weeks after the initial measurement and >15% relative decline in global longitudinal strain (GLS) compared with baseline strain (36, 40). In recognition of the limitation regarding evaluation of RV and to promote a more uniformed approach, the ASE published an updated guideline detailing the methodology (41).

### Right ventricular parameters

Results from one of the earlier trials investigating the effect of Anthracyclines on RV systolic and diastolic function using radionuclide angiography showed no significant change in RV EF during the one-year follow-up (42, 43). This is non-congruent to our own findings, which demonstrate a statistically significant drop in RV EF at follow-up. A study by Grover et al. using CMR to assess RV and LV function, not only concurred with our findings, demonstrating a decline in RV EF at 12-month follow-up but also, demonstrated a steeper decline in RV EF as compared to reduction in LVEF (20). Our meta-analysis established a statistically significant reduction in RVFAC, the parameter for RV radial systolic function among patients treated with Anthracyclines and/or Trastuzumab. This is on par with prior reports (19, 23, 26). Evaluation of RV strain is an important component of quantitating RV dysfunction, however it is a measurement that is subject to several methodological complexities. Consistent with our findings, several studies have demonstrated a significant decline in RVFWLS. Chang et al. (27) reported that RVFWLS may be a critical parameter for evaluation of occult RV dysfunction, suggestive of the increased susceptibility for damage within the thinner RV wall. Overall, among the studies that evaluated RV parameters, evaluation of strain may provide the most utility in early prediction of the effect of chemotherapy on the right ventricle. The discrepancies among the studies are most probably due to echocardiographic imaging techniques, as image acquisition and interobserver variability which may compromise the overall assessment. Additionally, the study population consisted of varying cardiovascular comorbidities, follow up periods and small sample sizes.

Despite the heterogeneity among the study population and the various parameters studied, there appears to be substantial evidence to suggest chemotherapy induced RV dysfunction. Larger studies with longer follow-up is necessary to confirm and establish the clinical impact and long term effects of these findings.

There is a growing body of evidence emphasizing the predictive significance of right ventricular structure and pathophysiology in different cardiac conditions (5, 6, 44–49). RV function is adversely affected even in untreated cancer patients, in part due to the abnormal physiology of increased pro-inflammatory markers, neurohumoral changes and circulating reactive oxidative species (47–49). However, there exists less evidence and guidance regarding the evaluation of right ventricle in patients undergoing chemotherapy. The implications of right ventricular dysfunction in cancer patients treated with Anthracycline and/or Trastuzumab have been reported in literature. Oliviera et al. (50) reported significant differences in the need for right ventricular assist devices in patients with chemotherapy associated cardiomyopathy, the majority of whom were treated with Anthracycline compared to all other causes of cardiomyopathy (19% vs. 9.3%, *p *< 0.0001), suggesting that RV dysfunction likely occurs in parallel with the more defined left ventricular dysfunction in this patient population (50).

Milano et al. (51) demonstrated a decrease in RV and LV thickness among mice infused with Doxorubicin or a combination of Doxorubicin and Trastuzumab. Both groups expressed increased RV free wall fibrosis in contrast to placebo or mice treated with Trastuzumab alone (51). Thus, it is intuitive that RV structure and function might be compromised following chemotherapy in human beings.

### Strengths and study limitations

The present meta-analysis has several limitations. First, this is a study level meta-analysis. Study level meta-analyses are limited in their ability to identify inter study heterogeneity. The dose of Anthracyclines and/or Trastuzumab used varied across studies. However, this has not been accounted for in the present analysis. All studies included in the present analysis had a follow-up period of less than 12 months. This was planned to avoid selection bias of survivors and because Anthracyclines and/or Trastuzumab induced cardiotoxicity is reversible. The study by Cottin et al., included both breath cancer and lymphoma patients. Finally, the present analysis did not assume any correlation between the pre and post treatment mean, so that a conservative pooled estimate with a wide confidence interval is obtained.

## Conclusive statements

In conclusion, our meta-analysis establishes that Anthracyclines and/or Trastuzumab significantly impact RV function as assessed by ecocardiography. While the impact on left ventricular function has long been recognised, our findings indicate that a comprehensive assessment should include right ventricular function. The implications of RV dysfunction in the context of CTRCD could be far-reaching, potentially affecting treatment decisions and patient prognosis. Future clinical guidelines and research should focus on this aspect, paving the way for improved cardiovascular care in patients undergoing chemotherapy. Our study underlines the need for larger studies with extended follow-ups to further confirm the clinical significance and long-term effects of RV dysfunction in this population.

## Data Availability

The original contributions presented in the study are included in the article/**Supplementary Material**, further inquiries can be directed to the corresponding author.
