# Supplementary material for: Chemotherapy induced right ventricular cardiomyopathy; a systematic review and meta-analysis
Source: Front Cardiovasc Med. 2023 Aug 3;10:1103941. doi: 10.3389/fcvm.2023.1103941 (PMC10434797; doi:10.3389/fcvm.2023.1103941)
Supplement: Supplementary file 1 [file Table4.docx]

| Database | Search details | Articles retrieved |
| --- | --- | --- |
| PubMed | (((((("epirubicin"[MeSH Terms] OR "epirubicin"[All Fields]) OR "epirubicin"[MeSH Terms]) OR "epirubicin"[MeSH Terms]) OR (("anthracyclines"[MeSH Terms] OR "anthracyclines"[All Fields] OR "anthracycline"[All Fields]) OR "anthracyclines"[MeSH Terms])) OR ((((("doxorubicin"[MeSH Terms] OR "doxorubicin"[All Fields]) OR "doxorubicin"[MeSH Terms]) OR "doxorubicin"[MeSH Terms]) OR (4[All Fields] AND epi[All Fields] AND "doxorubicin"[MeSH Terms])) AND ("epirubicin"[MeSH Terms] OR "epirubicin"[All Fields] OR ("4'"[All Fields] AND "epi"[All Fields] AND "doxorubicin"[All Fields]) OR "4' epi doxorubicin"[All Fields]))) OR ("trastuzumab"[MeSH Terms] OR "trastuzumab"[All Fields])) AND ((((((("cardiotoxicity"[MeSH Terms] OR "cardiotoxicity"[All Fields]) OR "myocardial strain"[All Fields]) OR "cardiotoxins"[MeSH Terms]) OR ("heart ventricles"[MeSH Terms] OR ("heart"[All Fields] AND "ventricles"[All Fields]) OR "heart ventricles"[All Fields] OR "ventricular"[All Fields])) OR "ventricular failure"[All Fields]) OR "Ejection fraction"[All Fields]) AND "stroke volume"[MeSH Terms]) | 521 |
| Cochrane Central Register of Controlled Trials | [epirubicin[MeSH Terms] OR anthracyclines[MeSH Terms] OR  doxorubicin"[MeSH Terms] ] AND [cardiotoxicity"[MeSH Terms] OR “myocardial strain” [MeSH Terms] OR ventricular [MeSH Terms]] | 1151 |

**Supplementary Table 1**: Search strategy
